# Combined PRP and CCP Therapy Suppresses Inflammation and Protects Cartilage in Post-Traumatic Osteoarthritis

**DOI:** 10.3390/vetsci13060506

**Published:** 2026-05-22

**Authors:** Tianwen Ma, Yongti Liu, Yanan Li, Hui Bai, Xiaxin Liu, Zongsheng Qiu, Yuhui Ma, Hai Li, Baoming Shi

**Affiliations:** 1 National Postdoctoral Research Workstation, Zhaosu Xiyu Horse Industry Co., Ltd., Yili 835699, China; 2 College of Animal Science and Technology, Northeast Agricultural University, Harbin 150030, China; 3 Heilongjiang Provincial Key Laboratory of Pathogenic Mechanism for Animal Disease and Comparative Medicine, Northeast Agricultural University, Harbin 150030, China; 4 College of Veterinary Medicine, Inner Mongolia Agricultural University, Hohhot 010010, China

**Keywords:** osteoarthritis, platelet-rich plasma, Cervus and Cucumis polypeptide, chondroprotection

## Abstract

This study aimed to evaluate the therapeutic effects of platelet-rich plasma (PRP) and Cervus and Cucumis polypeptide (CCP) injections in rats with post-traumatic osteoarthritis (OA). The model was established by transection of the anterior cruciate ligament, and the animals were subsequently treated with PRP and CCP. Articular cartilage degeneration was assessed through gross morphological observation, histopathological staining, and a standardized scoring system. Concurrently, pain-related behaviors, joint swelling, levels of inflammatory cytokines, and markers associated with extracellular matrix degradation were measured. The results demonstrated that, compared with the OA model group, PRP and CCP exhibited varying degrees of functional improvement, specifically, a reduction in pain-related behaviors and an alleviation of joint swelling. Furthermore, cartilage morphological damage was diminished, inflammatory marker levels decreased, and indicators of extracellular matrix degradation were attenuated. Histopathological examination of liver and kidney tissues revealed no apparent abnormalities. This study provides valuable experimental evidence for further treatment strategies for OA.

## Simple Summary

Osteoarthritis (OA) is a common joint disease in both animals and humans. Current treatments have limited efficacy, highlighting the need for new therapeutic approaches. Regenerative therapies, such as platelet-rich plasma (PRP), a platelet-rich biological preparation obtained from autologous blood by centrifugation, and traditional Chinese medicine-derived products, such as Cervus and Cucumis polypeptide injection (CCP, marketed as Songmeile^®^), have shown promise in treating joint diseases. However, previous studies have mostly focused on the treatment of PRP or CCP alone. In this study, a post-traumatic OA model was established in rats by transecting the knee ligament. The effects of combined PRP and CCP treatment on pain-related behaviors, joint swelling, and inflammatory marker levels were then evaluated. This study provides valuable experimental evidence for further treatment strategies for OA.

## 1. Introduction

Osteoarthritis (OA) is one of the most common chronic musculoskeletal diseases in animals and is widely observed in species such as dogs, horses, and cattle. Among lame horses, up to 60% of cases are associated with OA. Similarly, approximately 23.9% of dogs undergoing long-term follow-up have been diagnosed with OA [1,2]. The pathological changes in OA are primarily characterized by cartilage degeneration, subchondral bone remodeling, and synovial inflammation. Consequently, affected joints exhibit pain, while the limbs and hooves develop deformity and lameness, severely compromising both animal welfare and production performance. Despite the high incidence of OA, there is a lack of clinical treatments capable of effectively halting or reversing cartilage degeneration. Early-stage treatment focuses on alleviating joint pain symptoms, whereas advanced cases primarily involve joint replacement surgery [3]. In recent years, biologic therapies have attracted increasing attention for the treatment of OA.

PRP offers significant advantages in the treatment of OA and the promotion of cartilage regeneration owing to its easy availability, low cost, and the presence of various growth factors required by tissues [4,5]. Multiple clinical data have confirmed that patients with knee OA who receive PRP treatment experience significant improvements in joint function and effective pain relief, demonstrating superior clinical efficacy compared to traditional sodium hyaluronate treatment [6,7]. Recent studies have found that PRP treatment for knee OA can significantly improve joint function and relieve pain, and its therapeutic effect is related to platelet concentration [8,9,10]. Wu Q et al. found that weekly intra-articular injection of 50 μL of PRP in OA rats for 6 consecutive weeks reduced cartilage degeneration and inflammation in experimental knee OA, an effect that may be related to PRP-mediated regulation of the OPG/RANKL/RANK pathway [11]. Current research mainly focuses on the effects of intra-articular PRP injection on pain and subchondral bone changes, while studies on cartilage structural changes and related mechanisms are relatively few.

Cervus and Cucumis polypeptide injection (CCP, Songmeile^®^) is a peptide-based commercial preparation approved in China and, derived from acid-hydrolyzed, peptide-enriched extracts of sika deer (*Cervus nippon* Temminck) bone and aqueous extracts of muskmelon (*Cucumis melo* L.) seeds [12]. CCP has been reported to regulate bone metabolism, stimulate osteoblast activity, promote fracture healing, and exert anti-inflammatory or analgesic effects in orthopedic and inflammatory disease settings [13,14,15]. In experimental arthritis, CCP reduced paw edema and bone destruction and inhibited osteoclast differentiation [14]. Nevertheless, most of the available evidence on CCP relates to bone metabolism and fracture healing, rather than post-traumatic OA. Its mechanism of action and efficacy in OA therefore remain incompletely defined.

Although PRP and CCP have each been investigated in orthopedic or joint-related disorders, their potential combined use in post-traumatic OA has not been adequately evaluated. While PRP and CCP exert their effects through distinct pathways, they may exhibit complementary or additive interactions. Therefore, this study aimed to evaluate the effects of combined versus individual administration of PRP and CCP in a rat model of post-traumatic OA. The assessment encompassed their regulatory effects on pain, articular cartilage degeneration, histopathological features, ECM degradation, inflammatory cytokines, and OA-related biomarkers. We hypothesized that combined PRP and CCP treatment may exert complementary effects by reducing intra-articular inflammation and preserving cartilage matrix homeostasis.

## 2. Materials and Methods

Experimental animals: Power analysis was performed with α = 0.05 and statistical power = 0.80, indicating that at least 10 rats per group were required to detect a biologically meaningful difference among groups. Fifty healthy male Sprague Dawley rats (5–6 weeks old, 250–300 g) were used in this study. After acclimatization, 50 rats were randomly allocated to five groups by an investigator who was not involved in treatment administration, outcome assessment, or data analysis. Rats were numbered before grouping, and then randomly assigned to different experimental groups using a random number table method. All rats were housed under a 14/10 h light/dark cycle with adequate temperature, ventilation, food, and water. All procedures were approved by the research institution’s ethics committee (NEAUEC2024 03 48). During the experiment, no experimental rats were excluded. All were included in the final analysis.

CCP Sources: CCP was purchased from Harbin Yuheng Pharmaceutical Co., Ltd. in Harbin, China. Its primary constituents are the bones of the sika deer (*Cervus nippon* Temminck, family Cervidae) and the dried, mature seeds of the melon (*Cucumis melo* L., family Cucurbitaceae). The main constituents include bone-inducing polypeptides, various free amino acids, and active factors such as calcium and phosphorus [12,13,14,15].

Blinding Procedures: The personnel responsible for administering injections were not blinded to group assignments; however, they did not participate in outcome assessment or data analysis. The outcome assessors responsible for behavioral testing, gross morphological scoring, histological evaluation, quantitative immunohistochemical analysis, and biomarker assays were blinded to group assignments. Data analysts were also blinded to the group codes until the statistical analysis was completed.

Experimental Design and Grouping: Rats were randomly assigned to five groups (Control, OA, PRP, CCP, and CCP + PRP), with 10 rats in each group. Post-traumatic OA was induced by anterior cruciate ligament transection (ACLT), while the Control group received sham surgery (only the joint capsule was cut). The drawer test was used to verify successful OA modeling [16]. After ACLT or sham surgery, rats were placed individually in clean cages and kept on a warming pad until full recovery from anesthesia. Animals were allowed to move freely and had ad libitum access to food and water after recovery. Postoperative analgesia was provided with buprenorphine immediately after surgery and then every 12 h for 24–48 h. Analgesic administration was limited to the immediate postoperative period and was discontinued before formal behavioral testing to minimize potential interference with pain-related outcome measures. Two weeks after ACLT, the PRP group was administered intra-articular PRP (50 μL, once weekly for 8 weeks); the CCP group received intraperitoneal CCP (1.67 mg/kg) for 8 weeks; the combined group was given both treatments. The Control and OA groups were treated with 0.9% NaCl solution.

Allogeneic PRP Collection and Preparation: Allogeneic PRP was prepared from healthy Sprague Dawley donor rats of the same age and body weight range as the experimental animals. Donor rats were not included in the treatment or outcome assessment groups. Blood collection was performed under general anesthesia using isoflurane inhalation. After the absence of response to toe pinch was confirmed, whole blood was collected by cardiac puncture using sterile syringes containing anticoagulant. The whole blood was first centrifuged at 215 *g* for 10 min, and the plasma above the buffy coat was collected. Subsequently, a second centrifugation was performed at 863 *g* for 10 min to obtain PRP, with the supernatant platelet-poor plasma (PPP) discarded [17]. The platelet concentration was determined to be (1958.33 ± 316.41) × 10^9^/L using an automated hematology analyzer (XE-5000, Sysmex Medical Electronics (Shanghai) Co., Ltd., Shanghai, China).

Sample collection and processing: Rat synovial fluid was collected from weeks 2 through 10 for the detection of inflammatory factors and COMP. The experiment ended 10 weeks after ACLT surgery (8 weeks of continuous PRP intervention), and rats in each group were euthanized. The knee joint cartilage morphology was observed, and the knee joint cartilage was prepared for pathological sections and immunohistochemical analysis.

Joint Swelling Assessment: Joint width was measured with electronic calipers. Right knee joint width was measured before surgical modeling and then weekly after pharmacological intervention until the end of the experiment.

Von-Frey mechanical pain detection: The soles of the feet of each group of rats were stimulated using calibrated Von-Frey mechanical pricking filaments (0.4, 0.6, 1.0, 2.0, 4.0, 6.0, 8.0 and 15.0 g).

Cold Sensitivity Detection: After each rat was placed on a metal mesh floor and allowed to remain still for 15 min, acetone was applied from below the mesh to the center of the rat’s right hind paw. Behavioral changes were observed and scored within 20 s of acetone application.

Cartilage Morphology Observation and Pelletier Score: Knee joint samples were collected from each group of rats. Images of the femoral surface of the knee joint were taken using a Nikon D5300 AF-SD NIKKOR 18–55 mm SLR camera (Nikon Imaging Instruments Sales (China) Co., Ltd., Beijing, China) to observe changes in the surface morphology of the articular cartilage. The articular cartilage surface morphology changes were scored according to the Pelletier scoring system [18].

Enzyme-linked immunosorbent assay (ELISA): ELISA kits for IL-1β, TNF-α, and COMP were purchased from Nanjing Jiancheng Bioengineering Institute, China. The absorbance (OD value) of each sample was measured using a microplate reader, and the concentration of each biomarker was calculated against a standard curve.

Statistical analysis: Continuous data were assessed for normality and homogeneity of variance and are presented as mean ± standard deviation. Data meeting parametric assumptions were analyzed using one-way ANOVA followed by Tukey’s post hoc test, whereas non-parametric data were analyzed using the Kruskal–Wallis test followed by Dunn’s multiple comparison test. Longitudinal repeated-measures outcomes were analyzed using mixed-effects models: Gaussian linear mixed-effects models for continuous outcomes and a negative binomial generalized linear mixed-effects model with a log link for cold hypersensitivity score. In all longitudinal models, treatment group, time, and group × time interaction were included as fixed effects, and animal ID was included as a random intercept. Post hoc pairwise comparisons of estimated marginal means were performed at each time point with Tukey adjustment. Pelletier and OARSI scores are presented as median with interquartile range and were analyzed using the Kruskal–Wallis test followed by Dunn’s multiple comparison test. A two-sided *p* < 0.05 was considered statistically significant. The results of the mixed-effects model analysis can be found in Tables S1–S4. Effect sizes and 95% confidence intervals were calculated where applicable.

## 3. Results

### 3.1. CCP and PRP Effectively Slow Down the Progression of Cartilage Degeneration

**Figure 1:**
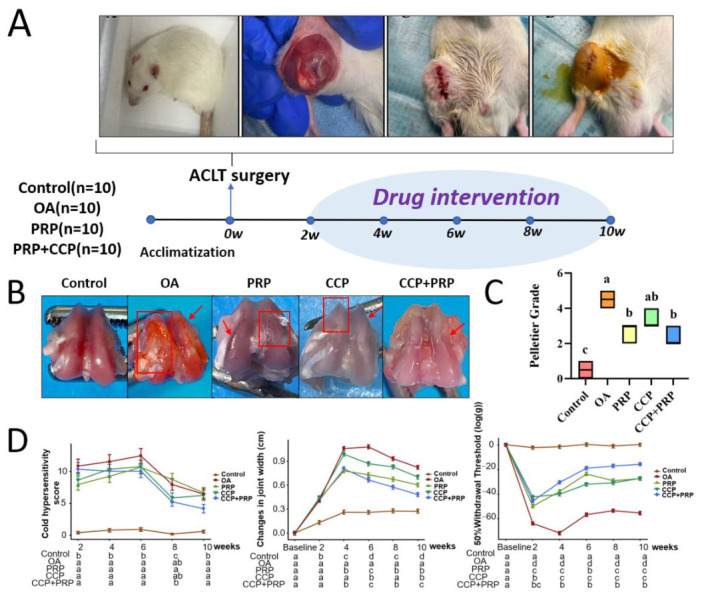
Effects of CCP, PRP, and combined CCP+PRP treatment on macroscopic cartilage morphology and functional outcomes in ACLT-induced OA. (**A**) Experimental design and treatment schedule. (**B**) Representative gross images of femoral articular cartilage. The red arrows and rectangles indicate areas of severe cartilage lesions. (**C**) Quantitative assessment of cartilage surface damage using the Pelletier scoring system. (**D**) Mechanical pain threshold, knee joint width, and cold sensitivity score during the experimental period. For longitudinal outcomes, statistical comparisons were based on estimated marginal means derived from mixed-effects models including group, time, and group × time interaction as fixed effects and animal ID as a random effect. Different lowercase letters at the same time point indicate significant differences among groups after Tukey adjustment, whereas the same lowercase letter indicates no significant difference, *p* < 0.05. N = 10 per group.

Ten weeks after ACLT surgery, the femurs of rats were grossly observed, and the degree of cartilage damage was scored using the Pelletier scoring method (Figure 1B,C). In the Control group, the cartilage surface was smooth and intact, with regular margins, and no surface defects or structural collapse were observed. In the OA group, the cartilage surface was rough and dull, with large areas of defects and fissures. In the PRP and CCP groups, the cartilage surface remained rough, but large cartilage defects were not observed. In the CCP + PRP group, no obvious cartilage defects were observed, although mild surface roughness remained. Compared with the Control group, the Pelletier grade was significantly higher in the OA group (*p* < 0.01), confirming gross cartilage damage after ACLT (Figure 1C). For change in knee joint width (F {20,225} = 68.91, *p* < 0.001) and change in mechanical pain threshold (F {20,225} = 62.20, *p* < 0.001), significant group × time interactions were observed, indicating that the longitudinal trajectories differed among treatment groups. At multiple time points between 4 and 10 weeks post-operatively, the combination therapy group tended to show greater improvement compared to the PRP and CCP monotherapy groups, with inter-group differences reaching statistical significance (*p* < 0.05). In contrast, cold hypersensitivity score showed a significant overall group effect (χ^2^ {4} = 45.53, *p* < 0.001), but neither the time effect nor the group × time interaction (χ^2^ {16} = 23.30, *p* = 0.106) reached statistical significance. Post hoc comparisons indicated that at most assessment time points, the differences observed among the various treatment groups were not statistically significant; only at Week 8 was the score in the CCP + PRP group significantly lower than that in the PRP group. Consequently, evidence suggesting that cold hypersensitivity symptoms exhibit treatment-dependent variations over time or that the combination therapy regimen offers a sustained advantage in alleviating cold hypersensitivity remains limited (Figure 1D; Tables S2–S4).

**Figure 2:**
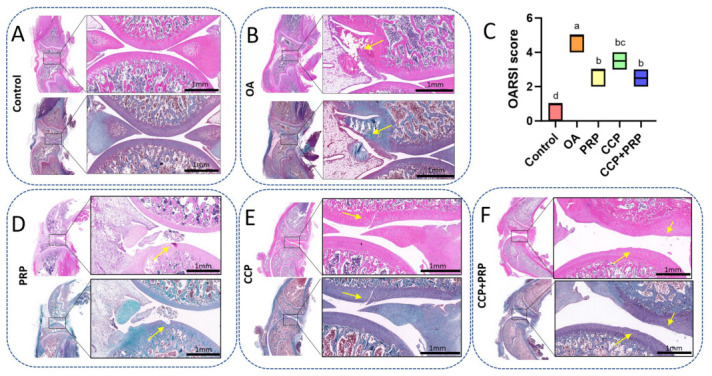
Histological assessment of articular cartilage degeneration in ACLT-induced OA. (**A**,**B**,**D**–**F**) Representative H&E and Safranin O–Fast Green staining images of knee articular cartilage from each experimental group. The yellow arrows indicate areas of severe cartilage damage. (**C**) Quantitative analysis of cartilage degeneration using the OARSI scoring system. Different lowercase letters indicate significant differences between groups, while the same lowercase letter indicates no significant difference (*p* < 0.05). N = 10 per group.

The degree of cartilage damage was assessed using H&E staining, Safranin O–Fast Green staining, and the OARSI scoring method. The histological findings were consistent with the gross pathological observations. In the Control group, the cartilage surface was smooth, chondrocytes were evenly distributed, matrix staining was preserved, and no obvious chondrocyte clusters were observed. In the OA group, the cartilage surface was rough, with ulceration foci, numerous chondrocyte clusters, superficial chondrocyte necrosis, and loss of matrix staining. Compared with the OA group, PRP and CCP treatment reduced OARSI scores and partially improved cartilage surface roughness, matrix staining loss, and chondrocyte clustering, although focal ulceration or cartilage fissures remained. The combined treatment group showed no obvious ulceration foci or cartilage fissures, with reduced cartilage surface roughness, chondrocyte cluster formation, and chondrocyte necrosis, as well as improved chondrocyte distribution and matrix staining. Compared with the OA group, the combined treatment group showed a significantly lower OARSI score, with a median difference of −2.0 points, 95% CI −3.0 to −2.0, and Cliff’s δ = −1.00 (Figure 2).

### 3.2. CCP and PRP Reduced Cartilage Matrix Degradation Markers

**Figure 3:**
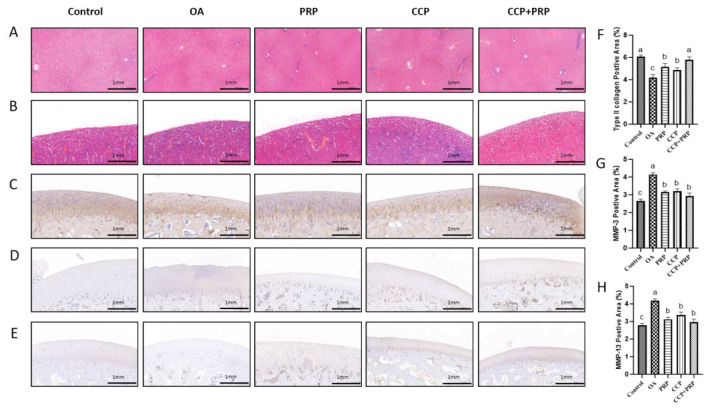
Effects of CCP, PRP, and combined treatment on cartilage matrix-related markers and liver and kidney morphology. (**A**) Representative H&E staining images of liver tissue. (**B**) Representative H&E staining images of kidney tissue. (**C**–**E**) Representative immunohistochemical staining images of type II collagen, MMP-3, and MMP-13 in femoral articular cartilage. (**F**–**H**) Semi-quantitative analysis of immunohistochemical staining for type II collagen, MMP-3, and MMP-13. Different lowercase letters indicate significant differences between groups, while the same lowercase letter indicates no significant difference (*p* < 0.05). N = 10 per group.

Immunohistochemistry was used to detect the effects of CCP, PRP, or combined treatment on the degradation level of cartilage matrix in each group. Results showed that, except for the control group, the expression levels of MMP-3 and MMP-13 in cartilage tissue were significantly increased, while the expression level of type II collagen was significantly decreased in all groups. Each treatment group showed significantly reduced expression levels of MMP-3 and MMP-13 compared with the OA group, suggesting attenuation of cartilage matrix degradation (Figure 3C–H). H&E staining was performed to examine whether obvious histopathological abnormalities were present in liver and kidney tissues following CCP, PRP, or combined treatment. Results showed that the liver tissue of rats in each group was intact with clear boundaries, hepatocytes were arranged radially, the cytoplasm was uniformly stained and the nuclei had normal morphology, and no inflammatory infiltration was observed in the portal area (Figure 3A). The kidney tissue showed intact renal corpuscle structure, uniform distribution of glomeruli, clear Bowman’s capsule without exudation, tightly arranged renal tubules of all levels with intact epithelial cell structure, and no abnormal proliferation of interstitium (Figure 3B). These observations indicate that no apparent histopathological evidence of liver or kidney injury was identified in the examined tissues. However, systemic toxicity was not comprehensively evaluated because serum biochemical indicators and other organ-function assessments were not included.

### 3.3. CCP and PRP Reduce Inflammatory Factors and Cartilage Matrix Degradation Levels

Following ACLT surgery, the levels of inflammatory factors (IL-1β and TNF-α) in rats were consistently higher than those in the control group, peaking at weeks 6–8. Mixed-effects model analysis revealed significant “Group × Time” interactions for IL-1β (F {16,180} = 26.82, *p* < 0.001), TNF-α (F {16,180} = 25.86, *p* < 0.001), and COMP (F {16,180} = 90.43, *p* < 0.001), indicating that the changes in these biomarkers exhibited time-dependent dynamics influenced by the treatment modality (Tables S3 and S4).

**Figure 4:**
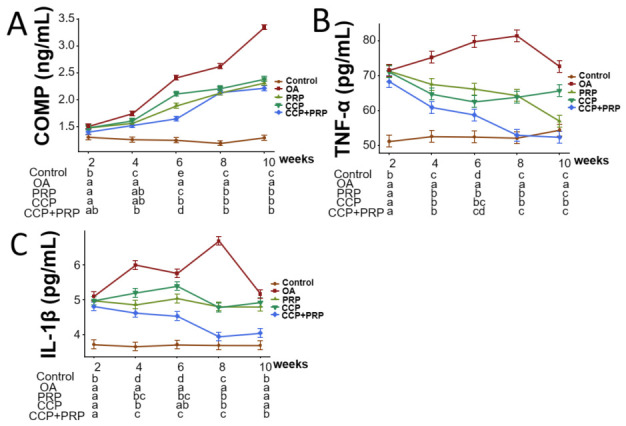
Effects of CCP, PRP, and combined treatment on inflammatory cytokines and COMP levels in ACLT-induced OA. (**A**) COMP levels in synovial fluid during the experimental period. (**B**) TNF-α levels in synovial fluid during the experimental period. (**C**) IL-1β levels in synovial fluid during the experimental period. For longitudinal outcomes, statistical comparisons were based on estimated marginal means derived from mixed-effects models including group, time, and group × time interaction as fixed effects and animal ID as a random effect. Different lowercase letters at the same time point indicate significant differences among groups after Tukey adjustment, whereas the same lowercase letter indicates no significant difference, *p* < 0.05. N = 10 per group.

Post hoc analysis demonstrated that the combined therapy group exhibited significantly lower levels of IL-1β than the CCP group at weeks 4, 6, 8, and 10, and significantly lower levels than the PRP group at weeks 8 and 10. TNF-α levels in the CCP+PRP group were significantly lower than those in the PRP group at weeks 6 and 8 and lower than those in the CCP group at weeks 8 and 10. Regarding COMP, levels in the combined therapy group were significantly lower than those in both monotherapy groups at week 6; however, at subsequent time points, the differences between groups did not reach statistical significance (Figure 4).

## 4. Discussion

In this study, we systematically evaluated the protective effects of CCP, PRP, and their combination against cartilage degeneration in a rat model of ACLT-induced OA. The results demonstrated that both CCP and PRP effectively alleviated the progression of cartilage degeneration in rat knee OA. Their effects are primarily manifested as improvements in functional outcomes, a reduction in intra-articular inflammation levels (IL-1β, TNF-α), and decreased expression of enzymes associated with cartilage matrix degradation (MMP-3, MMP-13). No obvious histopathological changes were observed in the liver or kidney tissues of rats following CCP or PRP treatment.

Previous studies have confirmed that cartilage degeneration is an important pathological manifestation in the progression of OA, and cartilage matrix degradation and chronic intra-articular inflammation are key factors driving cartilage degeneration [19]. Simultaneously, the massive release of inflammatory factors mediates pain sensitization, triggering clinical symptoms such as joint pain and swelling, becoming a significant contributing factor to OA functional impairment [20]. PRP, a widely studied orthobiologic, primarily repairs damaged tissue by exogenously supplementing components such as growth factors, cytokines, and platelet microparticles [21,22]. Furthermore, growth factors can effectively block the intra-articular inflammatory cascade by inhibiting the synthesis of pro-inflammatory cytokines in synovial fibroblasts, chondrocytes, and osteoblasts [22,23]. CCP, on the other hand, promotes repair by regulating the expression levels of key family members such as bone morphogenetic proteins (BMPs), transforming growth factor-β, and fibroblast growth factor. It also exerts anti-inflammatory effects by regulating the expression of key factors in inflammatory pathways [24]. Although both PRP and CCP can promote tissue repair by increasing the levels of intra-articular bioactive substances, they differ significantly in the composition and pathways of action of these bioactive substances. This study used an ACLT rat model of OA as the research subject to explore the therapeutic value of CCP and PRP injections from multiple dimensions, including cartilage morphology, functional improvement, inflammation regulation, and matrix protection.

This study evaluated the alleviating effects of CCP and PRP on cartilage degeneration through gross morphological observation, Pelletier score, Safranin O–Fast Green Staining, H.E. staining, and OARSI score. The results showed that pathological cartilage damage was significantly reduced in all treatment groups, with significantly lower Pelletier and OARSI scores than the OA group. Interestingly, the Pelletier and OARSI scores in the combination therapy group were lower than those in the single-treatment group, but the difference was not statistically significant.

Pain relief and joint function improvement are crucial needs in the clinical treatment of OA. The occurrence of pain and functional impairment is closely related to intra-articular inflammation-mediated pain sensitization and joint swelling. In this study, all treatment groups showed improvements in OA-associated mechanical pain, cold hypersensitivity, and joint swelling. The combined treatment group showed numerically greater improvement in some functional outcomes; however, this advantage over monotherapy was not consistently significant across all time points. This result may be correlated with the regulatory effect of intra-articular inflammatory factors. Bo Liao et al. reported that PRP-derived exosomes reduced pain behavior and inflammation in DMM mice by activating the PI3K/Akt signaling pathway in synovial endothelial cells, thereby enhancing synovial lymphatic function and promoting the clearance of inflammatory cells and related cytokines [25]. Guangyu Du found that PRP can also inhibit inflammation and apoptosis through the Nrf2/HO-1 pathway, slowing OA progression [17]. Huajun Huang et al. found that CCP can effectively reduce the expression levels of inflammatory factors in rabbit joints [26]. Researchers have also found that *Cervus nippon* Temminck, a component of CCP, can improve inflammation and oxidative stress caused by OA by regulating NLRP3 inflammatory cell pyroptosis and the Nrf2/HO-1/NQO1 signaling pathway, thereby delaying the progression of OA [27]. In the present study, both the PRP and CCP monotherapy groups also exhibited marked inhibitory effects on intra-articular inflammation, as evidenced by significantly reduced IL-1β and TNF-α levels, and effectively attenuated cartilage matrix degradation.

Disruption of cartilage matrix homeostasis is a core molecular mechanism of cartilage degeneration. Overexpression of matrix-degrading enzymes, coupled with reduced synthesis of cartilage matrix components, collectively leads to structural damage and functional loss of cartilage. Researchers such as Liang Cheng et al. found that PRP can inhibit the PI3K/AKT/mTOR pathway, downregulate inflammatory factors, and reduce the expression of cartilage matrix-degrading enzymes in cartilage [28]. In this study, all treatment groups significantly inhibited the expression of MMP-3 and MMP-13 and reduced COMP levels, confirming that both CCP and PRP can maintain matrix homeostasis and delay cartilage degeneration by regulating cartilage matrix degradation and synthesis.

This study represents an investigation to compare the overall therapeutic efficacy of combined CCP and PRP injection in a rat model of OA. Nevertheless, this study is subject to certain limitations. Specifically, the study utilized only a rat model of post-traumatic OA induced by ACLT. Although this model is widely employed to investigate cartilage degeneration and inflammatory responses associated with OA, it cannot fully replicate the complex pathological processes of naturally occurring OA observed in clinical veterinary cases. Therefore, further validation is required in various animal models of OA. The saline-treated OA group served as a disease control to account for potential spontaneous or natural recovery in the ACLT model; however, time-dependent adaptation cannot be completely excluded, particularly for behavioral outcomes. CCP is a region-specific commercial product, and its composition, manufacturing consistency, regulatory approval, and clinical availability may vary across regions. Furthermore, the specific molecular targets and signaling pathways involved and the presence of any dose-dependent protective effects require further elucidation through in vitro cellular experiments and molecular mechanism studies. In addition, although platelet concentration was measured, PRP was not comprehensively characterized with respect to leukocyte content, erythrocyte contamination, platelet activation status, or growth factor/cytokine composition. Furthermore, systemic safety assessment was limited to histopathological observation of liver and kidney tissues. This study did not test serum biochemical indicators, hematological parameters, or broader toxicological parameters of liver and kidney function, but no significant abnormalities were observed in pathological examination.

## 5. Conclusions

In summary, this study demonstrates that CCP and PRP treatments can alleviate ACLT-induced cartilage degeneration in a rat model of OA. These therapeutic effects were reflected in the reduction in inflammatory cytokine levels, inhibition of cartilage matrix degradation, and maintenance of cartilage matrix homeostasis. These findings provide experimental evidence for further research on CCP and PRP treatment of OA.

## Data Availability

The original contributions presented in this study are included in the article/Supplementary Material. Further inquiries can be directed to the corresponding authors.

## Supplementary Materials

The following supporting information can be downloaded at: https://www.mdpi.com/article/10.3390/vetsci13060506/s1, Table S1: Mixed-Effects Model Structure and Fit for Each Outcome Variable. Table S2: Type III ANOVA for Each Outcome Variable (Fixed Effects). Table S3: Estimated Marginal Means (EMM) of Outcome Variables by Group × Time. Table S4: Post-hoc Pairwise Comparisons of 5 Groups at the Same Time Point.

## Abbreviations

The following abbreviations are used in this manuscript:

OA	Osteoarthritis
PRP	Platelet-rich plasma
CCP	Cervus and Cucumis polypeptide injection
ECM	Extracellular matrix
ACLT	Anterior cruciate ligament transection
PPP	Platelet-poor plasma
