# Supplementary material for: Combined PRP and CCP Therapy Suppresses Inflammation and Protects Cartilage in Post-Traumatic Osteoarthritis
Source: Vet Sci. 2026 May 22;13(6):506. doi: 10.3390/vetsci13060506 (PMC13307649; doi:10.3390/vetsci13060506)
Supplement: Supplementary file 1 [file vetsci-13-00506-s001.zip › vetsci-4286204-supplementary.pdf]

Supplementary Materials

Table S1. Mixed-Effects Model Structure and Fit for Each Outcome Variable

Outcome Variable	Model Family	Fixed Effects	Random Effects	Number of Observations	Number of Animals	ICC
Change in Knee Joint Width (cm)	LMM (Gaussian; Heteroscedasticity by Group)	Group + Time + Group × Time	(1 ∣ animal_id)	300	50	0.681
Cold Hypersensitivity Score (Counts)	GLMM-NB2 (log)	Group + Time + Group× Time	(1 ∣ animal_id)	250	50	0.001
Change in Mechanical Pain Threshold (%)	LMM (Gaussian; Heteroscedasticity by Group)	Group + Time + Group × Time	(1 ∣ animal_id)	300	50	0.071
IL-1β (pg/mL)	LMM (Gaussian; Heteroscedasticity by Group)	Group + Time + Group × Time	(1 ∣ animal_id)	250	50	0.744
TNF-α (pg/mL)	LMM (Gaussian; Heteroscedasticity by Group)	Group + Time + Group × Time	(1 ∣ animal_id)	250	50	0.734
COMP (ng/mL)	LMM (Gaussian; Heteroscedasticity by Group)	Group + Time + Group × Time	(1 ∣ animal_id)	250	50	0.663

Note: ICC represents the proportion of variance attributable to differences between animals relative to the total variance; the ICC for GLMM-NB2 is based on the latent variable approach (residual variance = $\pi^2/3$).

Table S2. Type III ANOVA for Each Outcome Variable (Fixed Effects)

Outcome Variable	Source of Effect	Statistic	Value	Degrees of Freedom	**p**
Change in Knee Joint Width (cm)	Group	F	0.00	4, 45	1.000
Change in Knee Joint Width (cm)	Time	F	79.71	5, 225	<0.001***
Change in Knee Joint Width (cm)	Group × Time	F	68.91	20, 225	<0.001***
Cold Hypersensitivity Score (Counts)	Group	χ^2	45.53	4	<0.001***
Cold Hypersensitivity Score (Counts)	Time	χ^2	5.06	4	0.282
Cold Hypersensitivity Score (Counts)	Group × Time	χ^2	23.30	16	0.106
Change in Mechanical Pain Threshold (%)	Group	F	0.00	4, 45	1.000
Change in Mechanical Pain Threshold (%)	Time	F	0.72	5, 225	0.608
Change in Mechanical Pain Threshold (%)	Group × Time	F	62.20	20, 225	<0.001***
IL-1β (pg/mL)	Group	F	20.97	4, 45	<0.001***
IL-1β (pg/mL)	Time	F	0.16	4, 180	0.958
IL-1β (pg/mL)	Group × Time	F	26.82	16, 180	<0.001***
TNF-α (pg/mL)	Group	F	26.64	4, 45	<0.001***
TNF-α (pg/mL)	Time	F	1.76	4, 180	0.139
TNF-α (pg/mL)	Group × Time	F	25.86	16, 180	<0.001***
COMP (ng/mL)	Group	F	3.74	4, 45	0.010*
COMP (ng/mL)	Time	F	3.26	4, 180	0.013*
COMP (ng/mL)	Group × Time	F	90.43	16, 180	<0.001***

Note: LMM (Kenward-Roger degrees of freedom) was used for continuous outcomes, and GLMM-NB2 (Wald χ^2) was used for count outcomes (cold hypersensitivity score); * p<0.05, ** p<0.01, *** p<0.001.

Table S3: Estimated Marginal Means (EMM) of Outcome Variables by Group × Time

S3.1 Change in Knee Joint Width (cm)

Group	0	2	4	6	8	10
Control	-0.00 (0.02)	0.13 (0.02)	0.26 (0.02)	0.26 (0.02)	0.28 (0.02)	0.27 (0.02)
OA	-0.00 (0.03)	0.41 (0.03)	1.06 (0.03)	1.08 (0.03)	0.93 (0.03)	0.82 (0.03)
PRP	0.00 (0.03)	0.43 (0.03)	0.78 (0.03)	0.73 (0.03)	0.68 (0.03)	0.61 (0.03)
CCP	0.00 (0.03)	0.44 (0.03)	0.99 (0.03)	0.87 (0.03)	0.83 (0.03)	0.70 (0.03)
CCP+PRP	-0.00 (0.03)	0.42 (0.03)	0.81 (0.03)	0.67 (0.03)	0.58 (0.03)	0.48 (0.03)

Note: Data are presented as EMM (SE); time unit: weeks; preoperative measurements are designated as 0.

S3.2 Cold Hypersensitivity Score (Count)

Group	2	4	6	8	10
Control	0.40 (0.20)	0.80 (0.28)	0.90 (0.30)	0.20 (0.14)	0.60 (0.24)
OA	10.88 (1.07)	11.58 (1.10)	12.48 (1.15)	7.99 (0.91)	6.49 (0.82)
PRP	7.98 (0.91)	9.28 (0.98)	10.68 (1.06)	8.78 (0.95)	6.69 (0.83)
CCP	8.68 (0.95)	10.38 (1.04)	10.78 (1.06)	5.89 (0.78)	6.29 (0.80)
CCP+PRP	10.38 (1.04)	10.08 (1.02)	10.08 (1.02)	5.29 (0.73)	4.19 (0.65)

Note: Data are presented as EMM (SE); time unit: weeks.

S3.3 Change in Mechanical Pain Threshold (%)

Group	0	2	4	6	8	10
Control	0.00 (1.26)	-2.19 (1.26)	-1.52 (1.26)	0.30 (1.26)	-0.97 (1.26)	0.21 (1.26)
OA	0.00 (1.34)	-63.97 (1.34)	-71.79 (1.34)	-56.84 (1.34)	-53.55 (1.34)	-55.44 (1.34)
PRP	0.00 (1.26)	-49.84 (1.26)	-37.83 (1.26)	-23.60 (1.26)	-29.02 (1.26)	-27.42 (1.26)
CCP	0.00 (1.62)	-42.46 (1.62)	-40.20 (1.62)	-32.08 (1.62)	-30.87 (1.62)	-27.27 (1.62)
CCP+PRP	-0.00 (1.36)	-45.74 (1.36)	-30.49 (1.36)	-18.95 (1.36)	-17.13 (1.36)	-15.67 (1.36)

Note: Data are presented as EMM (SE); time unit: weeks; preoperative measurements are designated as 0.

S3.4 IL-1β (pg/mL)

Group	2	4	6	8	10
Control	3.72 (0.12)	3.65 (0.12)	3.71 (0.12)	3.69 (0.12)	3.69 (0.12)
OA	5.10 (0.13)	6.00 (0.13)	5.76 (0.13)	6.69 (0.13)	5.16 (0.13)
PRP	4.97 (0.13)	4.86 (0.13)	5.04 (0.13)	4.81 (0.13)	4.80 (0.13)
CCP	4.98 (0.12)	5.20 (0.12)	5.39 (0.12)	4.78 (0.12)	4.92 (0.12)
CCP+PRP	4.81 (0.12)	4.62 (0.12)	4.53 (0.12)	3.94 (0.12)	4.04 (0.12)

Note: Data are presented as EMM (SE); time unit: weeks.

S3.5 TNF-α (pg/mL)

Group	2	4	6	8	10
Control	51.30 (1.69)	52.67 (1.69)	52.55 (1.69)	52.25 (1.69)	54.48 (1.69)
OA	71.59 (1.70)	75.27 (1.70)	79.75 (1.70)	81.40 (1.70)	72.67 (1.70)
PRP	71.28 (1.70)	67.51 (1.70)	66.19 (1.70)	64.34 (1.70)	57.01 (1.70)
CCP	71.08 (1.71)	64.77 (1.71)	62.59 (1.71)	63.92 (1.71)	65.74 (1.71)
CCP+PRP	68.33 (1.63)	60.95 (1.63)	58.80 (1.63)	53.01 (1.63)	52.42 (1.63)

Note: Data are presented as EMM (SE); time unit: weeks.

S3.6 COMP (ng/mL)

Group	2	4	6	8	10
Control	1.30 (0.04)	1.26 (0.04)	1.25 (0.04)	1.19 (0.04)	1.29 (0.04)
OA	1.51 (0.05)	1.75 (0.05)	2.41 (0.05)	2.62 (0.05)	3.35 (0.05)
PRP	1.48 (0.05)	1.57 (0.05)	1.89 (0.05)	2.13 (0.05)	2.32 (0.05)
CCP	1.49 (0.05)	1.62 (0.05)	2.12 (0.05)	2.21 (0.05)	2.39 (0.05)
CCP+PRP	1.40 (0.05)	1.53 (0.05)	1.65 (0.05)	2.13 (0.05)	2.21 (0.05)

Note: Data are presented as EMM (SE); time unit: weeks.

Table S4: Post-hoc Pairwise Comparisons of 5 Groups at the Same Time Point

S4.1 Knee Joint Width Change (cm)

Inter-group Comparison	0	2	4	6	8	10
Control vs OA	0.00 (*p*=1.000)	-0.28 (*p* < 0.001)***	-0.80 (*p* < 0.001)***	-0.82 (*p* < 0.001)***	-0.65 (*p* < 0.001)***	-0.55 (*p* < 0.001)***
Control vs PRP	-0.00 (*p*=1.000)	-0.30 (*p* < 0.001)***	-0.52 (*p* < 0.001)***	-0.47 (*p* < 0.001)***	-0.40 (*p* < 0.001)***	-0.33 (*p* < 0.001)***
Control vs CCP	-0.00 (*p*=1.000)	-0.31 (*p* < 0.001)***	-0.73 (*p* < 0.001)***	-0.61 (*p* < 0.001)***	-0.55 (*p* < 0.001)***	-0.43 (*p* < 0.001)***
Control vs (CCP+PRP)	-0.00 (*p*=1.000)	-0.28 (*p* < 0.001)***	-0.54 (*p* < 0.001)***	-0.41 (*p* < 0.001)***	-0.30 (*p* < 0.001)***	-0.21 (*p* < 0.001)***
OA vs PRP	-0.00 (*p*=1.000)	-0.02 (*p*=0.984)	0.28 (*p* < 0.001)***	0.35 (*p* < 0.001)***	0.26 (*p* < 0.001)***	0.22 (*p* < 0.001)***
OA vs CCP	-0.00 (*p*=1.000)	-0.03 (*p*=0.894)	0.07 (*p*=0.322)	0.21 (*p* < 0.001)***	0.10 (*p*=0.064)	0.12 (*p*=0.019)*
OA vs (CCP+PRP)	-0.00 (*p*=1.000)	-0.01 (*p*=1.000)	0.25 (*p* < 0.001)***	0.41 (*p* < 0.001)***	0.36 (*p* < 0.001)***	0.34 (*p* < 0.001)***
PRP vs CCP	-0.00 (*p*=1.000)	-0.01 (*p*=0.995)	-0.21 (*p* < 0.001)***	-0.14 (*p*=0.004)**	-0.15 (*p*=0.001)**	-0.10 (*p*=0.067)
PRP vs (CCP+PRP)	0.00 (*p*=1.000)	0.01 (*p*=0.996)	-0.02 (*p*=0.960)	0.06 (*p*=0.415)	0.10 (*p*=0.065)	0.12 (*p*=0.014)*
CCP vs (CCP+PRP)	0.00 (*p*=1.000)	0.03 (*p*=0.940)	0.18 (*p* < 0.001)***	0.20 (*p* < 0.001)***	0.25 (*p* < 0.001)***	0.22 (*p* < 0.001)***

Note: Data are presented as mean differences (Pre-group − Post-group) and Tukey HSD-adjusted p-values.

S4.2 Cold Hypersensitivity Score (Count)

Inter-group Comparison	2	4	6	8	10
Control vs OA	0.04 (*p* < 0.001)***	0.07 (*p* < 0.001)***	0.07 (*p* < 0.001)***	0.02 (*p* < 0.001)***	0.09 (*p* < 0.001)***
Control vs PRP	0.05 (*p* < 0.001)***	0.09 (*p* < 0.001)***	0.08 (*p* < 0.001)***	0.02 (*p* < 0.001)***	0.09 (*p* < 0.001)***
Control vs CCP	0.05 (*p* < 0.001)***	0.08 (*p* < 0.001)***	0.08 (*p* < 0.001)***	0.03 (*p* < 0.001)***	0.10 (*p* < 0.001)***
Control vs (CCP+PRP)	0.04 (*p* < 0.001)***	0.08 (*p* < 0.001)***	0.09 (*p* < 0.001)***	0.04 (*p* < 0.001)***	0.14 (*p* < 0.001)***
OA vs PRP	1.36 (*p*=0.236)	1.25 (*p*=0.525)	1.17 (*p*=0.777)	0.91 (*p*=0.974)	0.97 (*p*=1.000)
OA vs CCP	1.25 (*p*=0.537)	1.12 (*p*=0.933)	1.16 (*p*=0.812)	1.36 (*p*=0.402)	1.03 (*p*=1.000)
OA vs (CCP+PRP)	1.05 (*p*=0.997)	1.15 (*p*=0.858)	1.24 (*p*=0.525)	1.51 (*p*=0.146)	1.55 (*p*=0.186)
PRP vs CCP	0.92 (*p*=0.984)	0.89 (*p*=0.940)	0.99 (*p*=1.000)	1.49 (*p*=0.132)	1.06 (*p*=0.997)
PRP vs (CCP+PRP)	0.77 (*p*=0.413)	0.92 (*p*=0.980)	1.06 (*p*=0.994)	1.66 (*p*=0.033)*	1.59 (*p*=0.130)
CCP vs (CCP+PRP)	0.84 (*p*=0.748)	1.03 (*p*=1.000)	1.07 (*p*=0.990)	1.11 (*p*=0.981)	1.50 (*p*=0.259)

Note: Data in the table are presented as rate ratios (Pre-group / Post-group) and p-values adjusted using Tukey's HSD. For cold hypersensitivity, the baseline (wk 0) values for all animals were 0—indicating an absence of signal—and could not be fitted using the GLMM-NB log-link model; therefore, the analysis was fitted starting from Week 2.

4.3 Change in Mechanical Pain Threshold (%)

Inter-group Comparison	0	2	4	6	8	10
Control vs OA	-0.00 (p=1.000)	61.77 (p < 0.001)***	70.26 (p < 0.001)***	57.14 (p < 0.001)***	52.58 (p < 0.001)***	55.65 (p < 0.001)***
Control vs PRP	-0.00 (p=1.000)	47.64 (p < 0.001)***	36.31 (p < 0.001)***	23.91 (p < 0.001)***	28.06 (p < 0.001)***	27.63 (p < 0.001)***
Control vs CCP	-0.00 (p=1.000)	40.26 (p < 0.001)***	38.68 (p < 0.001)***	32.38 (p < 0.001)***	29.90 (p < 0.001)***	27.48 (p < 0.001)***
Control vs (CCP+PRP)	0.00 (p=1.000)	43.54 (p < 0.001)***	28.97 (p < 0.001)***	19.25 (p < 0.001)***	16.16 (p < 0.001)***	15.88 (p < 0.001)***
OA vs PRP	0.00 (p=1.000)	-14.13 (p < 0.001)***	-33.95 (p < 0.001)***	-33.23 (p < 0.001)***	-24.53 (p < 0.001)***	-28.02 (p < 0.001)***
OA vs CCP	0.00 (p=1.000)	-21.51 (p < 0.001)***	-31.59 (p < 0.001)***	-24.75 (p < 0.001)***	-22.68 (p < 0.001)***	-28.17 (p < 0.001)***
OA vs (CCP+PRP)	0.00 (p=1.000)	-18.23 (p < 0.001)***	-41.29 (p < 0.001)***	-37.88 (p < 0.001)***	-36.42 (p < 0.001)***	-39.77 (p < 0.001)***
PRP vs CCP	-0.00 (p=1.000)	-7.38 (p=0.007)**	2.37 (p=0.776)	8.48 (p=0.001)**	1.84 (p=0.896)	-0.15 (p=1.000)
PRP vs (CCP+PRP)	0.00 (p=1.000)	-4.10 (p=0.194)	-7.34 (p=0.002)**	-4.65 (p=0.107)	-11.89 (p < 0.001)***	-11.75 (p < 0.001)***
CCP vs (CCP+PRP)	0.00 (p=1.000)	3.28 (p=0.536)	-9.70 (p < 0.001)***	-13.13 (p < 0.001)***	-13.74 (p < 0.001)***	-11.60 (p < 0.001)***

Note: Data are presented as mean differences (Pre-group − Post-group) and Tukey HSD-adjusted p-values.

S4.4 IL-1β (pg/mL)

Inter-group Comparison	2	4	6	8	10
Control vs OA	-1.39 (p < 0.001)***	-2.35 (p < 0.001)***	-2.05 (p < 0.001)***	-3.00 (p < 0.001)***	-1.47 (p < 0.001)***
Control vs PRP	-1.26 (p < 0.001)***	-1.21 (p < 0.001)***	-1.33 (p < 0.001)***	-1.12 (p < 0.001)***	-1.11 (p < 0.001)***
Control vs CCP	-1.26 (p < 0.001)***	-1.55 (p < 0.001)***	-1.68 (p < 0.001)***	-1.09 (p < 0.001)***	-1.23 (p < 0.001)***
Control vs (CCP+PRP)	-1.09 (p < 0.001)***	-0.97 (p < 0.001)***	-0.82 (p < 0.001)***	-0.25 (p=0.609)	-0.35 (p=0.276)
OA vs PRP	0.13 (p=0.956)	1.14 (p < 0.001)***	0.72 (p=0.003)**	1.89 (p < 0.001)***	0.36 (p=0.311)
OA vs CCP	0.13 (p=0.952)	0.80 (p < 0.001)***	0.37 (p=0.256)	1.91 (p < 0.001)***	0.24 (p=0.671)
OA vs (CCP+PRP)	0.30 (p=0.476)	1.38 (p < 0.001)***	1.23 (p < 0.001)***	2.75 (p < 0.001)***	1.12 (p < 0.001)***
PRP vs CCP	-0.00 (p=1.000)	-0.34 (p=0.346)	-0.36 (p=0.294)	0.03 (p=1.000)	-0.12 (p=0.962)
PRP vs (CCP+PRP)	0.17 (p=0.889)	0.24 (p=0.670)	0.51 (p=0.055)	0.87 (p < 0.001)***	0.76 (p=0.001)**
CCP vs (CCP+PRP)	0.17 (p=0.870)	0.58 (p=0.014)*	0.86 (p < 0.001)***	0.84 (p < 0.001)***	0.88 (p < 0.001)***

Note: Data are presented as mean differences (Pre-group − Post-group) and Tukey HSD-adjusted p-values.

S4.5 TNF-α (pg/mL)

Inter-group Comparison	2	4	6	8	10
Control vs OA	-20.29 ($p < 0.001$)***	-22.59 ($p < 0.001$)***	-27.20 ($p < 0.001$)***	-29.15 ($p < 0.001$)***	-18.19 ($p < 0.001$)***
Control vs PRP	-19.98 ($p < 0.001$)***	-14.83 ($p < 0.001$)***	-13.65 ($p < 0.001$)***	-12.09 ($p < 0.001$)***	-2.53 (p=0.827)
Control vs CCP	-19.78 ($p < 0.001$)***	-12.09 ($p < 0.001$)***	-10.05 (p=0.001)**	-11.68 ($p < 0.001$)***	-11.27 ($p < 0.001$)***
Control vs (CCP+PRP)	-17.03 ($p < 0.001$)***	-8.28 (p=0.008)**	-6.25 (p=0.075)	-0.77 (p=0.997)	2.05 (p=0.904)
OA vs PRP	0.31 (p=1.000)	7.76 (p=0.019)*	13.55 ($p < 0.001$)***	17.06 ($p < 0.001$)***	15.66 ($p < 0.001$)***
OA vs CCP	0.51 (p=1.000)	10.50 ($p < 0.001$)***	17.15 ($p < 0.001$)***	17.48 ($p < 0.001$)***	6.92 (p=0.046)*
OA vs (CCP+PRP)	3.26 (p=0.641)	14.31 ($p < 0.001$)***	20.94 ($p < 0.001$)***	28.39 ($p < 0.001$)***	20.25 ($p < 0.001$)***
PRP vs CCP	0.21 (p=1.000)	2.74 (p=0.787)	3.60 (p=0.573)	0.42 (p=1.000)	-8.73 (p=0.006)**
PRP vs (CCP+PRP)	2.95 (p=0.721)	6.55 (p=0.058)	7.39 (p=0.024)*	11.33 ($p < 0.001$)***	4.59 (p=0.309)
CCP vs (CCP+PRP)	2.75 (p=0.772)	3.81 (p=0.495)	3.79 (p=0.501)	10.91 ($p < 0.001$)***	13.32 ($p < 0.001$)***

Note: Data are presented as mean differences (Pre-group − Post-group) and Tukey HSD-adjusted p-values. * $p < 0.05$, ** $p < 0.01$, *** $p < 0.001$.

S4.6 COMP (ng/mL)

Inter-group Comparison	2	4	6	8	10
Control vs OA	-0.21 (p=0.018)*	-0.49 ($p < 0.001$)***	-1.16 ($p < 0.001$)***	-1.43 ($p < 0.001$)***	-2.05 ($p < 0.001$)***
Control vs PRP	-0.18 (p=0.045)*	-0.31 ($p < 0.001$)***	-0.64 ($p < 0.001$)***	-0.94 ($p < 0.001$)***	-1.02 ($p < 0.001$)***
Control vs CCP	-0.18 (p=0.041)*	-0.36 ($p < 0.001$)***	-0.87 ($p < 0.001$)***	-1.02 ($p < 0.001$)***	-1.09 ($p < 0.001$)***
Control vs (CCP+PRP)	-0.10 (p=0.511)	-0.27 (p=0.001)**	-0.40 ($p < 0.001$)***	-0.94 ($p < 0.001$)***	-0.92 ($p < 0.001$)***
OA vs PRP	0.03 (p=0.992)	0.18 (p=0.060)	0.52 ($p < 0.001$)***	0.49 ($p < 0.001$)***	1.03 ($p < 0.001$)***
OA vs CCP	0.02 (p=0.997)	0.13 (p=0.300)	0.30 ($p < 0.001$)***	0.41 ($p < 0.001$)***	0.96 ($p < 0.001$)***
OA vs (CCP+PRP)	0.11 (p=0.513)	0.22 (p=0.015)*	0.76 ($p < 0.001$)***	0.49 ($p < 0.001$)***	1.13 ($p < 0.001$)***
PRP vs CCP	-0.01 (p=1.000)	-0.05 (p=0.935)	-0.22 (p=0.010)*	-0.08 (p=0.719)	-0.07 (p=0.819)
PRP vs (CCP+PRP)	0.08 (p=0.755)	0.04 (p=0.973)	0.24 (p=0.005)**	-0.00 (p=1.000)	0.10 (p=0.504)
CCP vs (CCP+PRP)	0.08 (p=0.717)	0.09 (p=0.654)	0.47 ($p < 0.001$)***	0.08 (p=0.747)	0.17 (p=0.082)

Note: Data are presented as mean differences (Pre-group − Post-group) and Tukey HSD-adjusted p-values. * $p < 0.05$, ** $p < 0.01$, *** $p < 0.001$.
